# Thermal, Structural, and Rheological Characterization of Waxy Starch as a Cryogel for Its Application in Food Processing

**DOI:** 10.3390/polym10040359

**Published:** 2018-03-23

**Authors:** Jonathan Coria-Hernández, Abraham Méndez-Albores, Rosalía Meléndez-Pérez, Marta Elvia Rosas-Mendoza, José Luis Arjona-Román

**Affiliations:** 1 National Autonomous University of Mexico-Superior Studies Faculty at Cuautitlan (UNAM-FESC), Doctorate Program in Animal Production and Health Sciences, Cuautitlan Izcalli 54714, Mexico; jonathancoria@outlook.com; 2 National Autonomous University of Mexico-Superior Studies Faculty at Cuautitlan (UNAM–FESC), Campus 4. Multidisciplinary Research Unit L14 (Food, Mycotoxins and Mycotoxicosis), Cuautitlan Izcalli 54714, Mexico; 3 National Autonomous University of Mexico-Superior Studies Faculty at Cuautitlan (UNAM–FESC), Campus 4. Multidisciplinary Research Unit L13 (Thermal and Structural Analysis of Materials and Foods), Cuautitlan Izcalli 54714, Mexico; melendez@unam.mx (R.M.-P.); merosas@unam.mx ((M.E.R.-M.)

**Keywords:** waxy starch, cryogel, heat capacity, pore size, rheology

## Abstract

Starch is the major component of cereal, pulses, and root crops. Starch consists of two kinds of glucose polymers, amylose and amylopectin. Waxy starch—with 99–100% amylopectin—has distinctive properties, which define its functionality in many food applications. In this research, a novel material was prepared through the cryogelification of waxy starch (WS) using four cycles of freezing and thawing in indirect contact with liquid nitrogen at −150 °C. Polyvinyl alcohol (PVA) was used as a reference. The cryogels were characterized using several validation methodologies: modulated differential scanning calorimetry (MDSC), scanning electron microscopy (SEM), rheology, and Fourier transform infrared (FTIR) spectroscopy with diffuse reflectance (DR). Based on the number of freeze–thaw cycles, significant changes were found (*P* < 0.05) showing important structural modifications as well as reorganization of the polymeric matrix. Two cryogelification cycles of the WS were enough to obtain the best structural and functional characteristics, similar to those of PVA, which has already been tested as a cryogel. From these results, it is concluded that WS has potential as a cryogel for application in food processing.

## 1. Introduction

Cryogels are an emerging class of biomaterial that have recently drawn attention because of the diverse applications they have in different research areas. Cryogels can be defined as: (1) colloidal systems formed during the cryoprecipitation of the blood plasma when it is cooled down to approximately 4 °C; (2) polymers or inorganic systems first produced by the sol–gel technology and then by lyophilization; and (3) synthetic and natural polymeric materials produced in a frozen solvent, usually water [1,2].

In this research, the produced cryogels belong to the third definition. This material is obtained by cryotropic gelation of polymeric precursors through homogeneous or heterogeneous network formation with physical stability and covalent cross-linking. At very low temperatures, most of the solvent freezes, while a portion is left to unfreeze (non-frozen liquid micro-phase). The dissolved substances are concentrated and undergo polymerization and structural rearrangement, which leads to the cryogel formation. Moreover, crystals act as an interconnected opening network which controls the pore size and generates mechanical resistance [1,2,3].

Cryogels are produced with a wide range of morphologies and properties, which can be adapted for particular applications. They are heterophasic non-transparent materials showing macropores (1 to 150 μm). Their properties depend on the pore structure (size, distribution and inter-connectivity), and on the thickness of the pore wall and density expressed as the concentration of the polymer in swollen pore walls. Mass transport inside the matrix is controlled by pore size and inter-connectivity. On the other hand, the thickness and density of the cryogel wall determine its mechanical properties.

The cryogels produced from bio-polymers significantly change their expansion degree after small modifications in the environmental factors, such as temperature, pH or ionic forces. The time response to mass and heat transfer is shorter with cryogels than with hydrogels [3].

One of the purposes of cryogels is the analysis of their application at low temperatures to biological systems and the use of this knowledge for the development of improved conservation protocols. The intra- and extracellular solute concentration, the constitution of permeable and impermeable solutes and the freeze–unfreeze rate are the three parameters that control the physicochemical conditions for the application of cryogels [1,2,3,4].

Polyvinyl alcohol (PVA) is a synthetic, non-toxic, water-soluble, biocompatible and biodegradable polymer that has been systematically studied due to its multiple characteristics, especially for several industrial and pharmaceutical applications such as films, emulsions and adhesives. Despite its synthetic composition, it is a semi-crystalline, hydrophilic polymer produced by the polymerization of vinyl acetate (PVAc) and its subsequent hydrolysis [5,6,7]. PVA applications are related to processes that involve energy changes; therefore, advanced research and analysis of the microstructure of the polymer as a function of temperature are very important for its use with cellular systems. However, its use in food processing is totally restricted, and the information could be used as a reference for the behavior of cryogels produced from other polymers [8].

Starch is a mixture of two glucose polymers, amylose and amylopectin [9]. Amylose is essentially a linear molecule consisting of α-(1,4)-linked *d*-glucopyranosyl units, while amylopectin, is a highly branched macromolecule consisting of α-(1,6)-linked *d*-glucopyranosyl units attached to α-(1,4)-bonds [10]. The amylose–amylopectin ratio differs among starches, but typical levels are 25–75% [11]. Some starches contain either higher amylose content (up to 70%) or higher amylopectin content (99–100%), such as waxy starch (WS). Compared to traditional starch, WS provides unique functions for diverse applications. It confers different properties such as the formation of more viscous and thermostable structures after freezing, with smooth texture, cohesivity, low opacity and thermo-reversibility in gels [12,13]. WS is preferably used in foods and the textile industry.

The objective of this work is the characterization of a WS cryogel to evaluate its structural, rheological and thermodynamic properties and to determine if there are important differences caused by the freeze–thaw process using PVA as a reference, as it has already been studied as a cryogel. 

## 2. Materials and Methods 

### 2.1. Sample Preparation

PVA (–CH_2_CHOH–_n_; Sigma-Aldrich, St. Louis, MO, USA, 146–186 kDa, 99% hydrolyzed in crystal) was dispersed in water (2.5% *w/w*) for 4 h at 80 °C with magnetic stirring [7]. WS (–C_6_H_10_O_5_–_n_; Firm-Tex, Ingredion, Mexico) was dispersed by magnetic stirring in water (3% *w/w*) at 40 °C for 2 h [14]. Freezing was carried out using the protocols described by Giannouli and Morris [14] and Lozinsky [15]. Briefly, the hydrogels were placed in cryogenic tubes, and then immersed in liquid nitrogen up to −150 ± 1 °C for 10 min; subsequently, tubes were thawed by immersion in water at 50 ± 2 °C for 30 min using four repeated cycles.

### 2.2. Thermal Analysis by Modulated Differential Scanning Calorimetry (MDSC)

Samples were analyzed using a differential scanning calorimeter with temperature modulation (DSC 2920, TA Instruments, New Castle, DE, USA). Cooling was carried out using a refrigerated cooling system (RCS). The temperature and heat capacity calibrations were performed using TA Instruments software with indium (melting point value of 156.6 °C) and sapphire (aluminum oxide), respectively. The TA Instruments universal analysis software (2000 V 4.5 A) was used to record and analyze all thermograms. Samples (4.5 ± 0.42 mg) were packed down in hermetic aluminum pans and were analyzed in triplicate by heating in the modulated DSC furnace at a rate of 5 °C/min with temperature modulation of 0.8 °C every 60 s. Nitrogen was used as a purge gas at a constant flow rate of 60 mL/min. Thermal decomposition data were collected over the temperature range of −50 to 150 °C [16,17].

### 2.3. Structural Ordering Degree

The structural ordering degree can be calculated by estimating the enthalpy of fusion from the area under the curve in MDSC endotherms by drawing a baseline from the first onset of the melting to the last trace of crystallinity using:

(1) $$X_{c}=\frac{∆H_{f}}{∆H_{f}^{0}}$$

where: $X_{c}$ is the weight fraction of crystallinity, $∆H_{f}$ is the enthalpy of fusion measured at the melting point *T*_m_, and $∆H_{f}^{0}$ is the enthalpy of fusion of the totally crystalline polymer dispersion measured at the equilibrium melting point $T_{m}^{0}$ [18].

### 2.4. Scanning Electron Microscopy (SEM)

The surface morphology and the microstructure of the cryogels were examined using an in-touch scope SEM (JEOL, JSM-6012LA, Tokyo, Japan). Samples were coated with a thin gold layer with a vacuum electric sputter coater (Denton Vacuum Inc., Desk V HP, Moorestown, NJ, USA) operated at 7 mA for 5 min to enhance the electron conductivity and image quality. Microscopy analysis was performed at 900× with an accelerating voltage of 7 kV under high vacuum.

### 2.5. Rheological Analysis

The rheological properties of the cryogels were measured by using a rotational viscosimeter (Rheomat RM180, Mapple Instruments, Toronto, ON, Canada) with attached computer software (RSI Orchestrator, Rheometrics Scientific, Piscataway, NJ, USA) at a programmed temperature of 25 ± 1.5 °C. A total of 25 mL of each sample were kept in a thermostatically controlled water bath for 10 min before being measured in order to attain a desirable temperature of 25 °C. First measurements were taken 2 min after the spindle was immersed in each sample, in order to allow thermal equilibrium in the sample. Each measurement was duplicated on the same sample. The shear rate versus shear stress data were interpreted using the power law expression (τ = k·γ^n^); where τ is the shear stress (N/m^2^), γ is the shear rate (s^−1^), n is the flow behavior index, and k is the consistency index (Ns^n^/m^2^). The values for the flow behavior index n, and the consistency index k were obtained from plots of log shear stress versus log shear rate, according to the power law equation (log τ = log k + n·log γ).

### 2.6. Fourier Transform Infrared-Diffuse Reflectance (FTIR-DR) Spectroscopy

The functional groups in the cryogels were characterized using a FTIR-DR Frontier SP8000 spectrophotometer (Perkin Elmer, Waltham, MA, USA) equipped with a deuterated triglycine sulfate (DTGS) detector and controlled with the Spectrum 10.4.2 software (Perkin Elmer Ltd, Bucks, UK). The ground samples (<250 µm) were placed in a diffuse reflectance (DR) sample holder and DR spectra were collected in a range of 400–4000 cm^−1^ at a resolution of 4 cm^−1^ by co-adding 32 scans. A background spectrum was obtained against air every day during the experiment. The spectra of both PVA and WS cryogels were collected in transmittance mode in quadruplicate and the average value was used.

### 2.7. Statistical Analysis

The experiment was conducted as a completely randomized design with three replicates. Data were assessed by one- and two-way analysis of variance (ANOVA) and means comparisons were performed according to the Tukey’s test using Minitab 16.0.1 software (Penn State University, Pennsylvania, PA, USA). A significance value of *P* < 0.05 was used to distinguish significant differences between treatments.

## 3. Results and Discussion

### 3.1. MDSC Analysis

**Figure 1:**
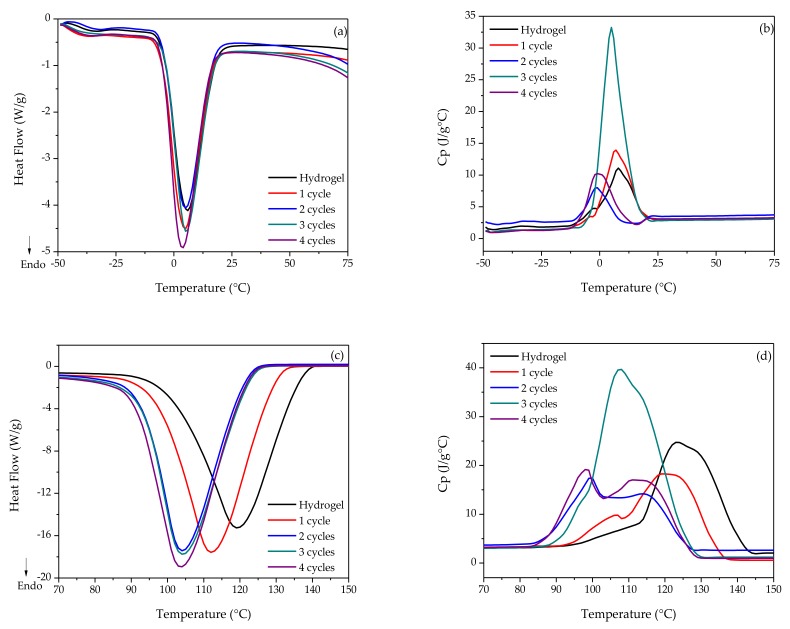
Thermal analysis of the hydro- and cryogels of PVA with different freeze–thaw cycles: (**a**) heat flow during melting; (**b**) fusion *C*p; (**c**) heat flow during evaporation; (**d**) evaporation *C*p.

The thermal behavior of the polymers was evaluated in order to determine changes in the enthalpy and transitions, indicating kinetic and structural changes after different freeze–thaw cycles with liquid nitrogen. For the PVA case (Figure 1a), the heat flow shows the same behavior, at an interval of −50 to 75 °C, presenting the same transitions with different magnitudes in enthalpy. The four-cycle cryogel has 345.0 J/g with a melting temperature (*T*_m_) of 3.62 °C showing higher enthalpy. This phenomenon is due to the existence of more crystallized water and therefore the need for more energy to carry out the fusion of the crystals [18,19,20,21].

Regarding the structural changes shown in the *C*p of Figure 1b, the fusion of PVA crystals generates important changes, showing that the cryogel at three cycles has major structural modifications due to molecular rearrangements after each cycle. As for the four-cycle cryogel case, important changes occurred in the same temperature range. According to El-Sayed et al. [19], the structural reorientation of the polymer molecules is higher after the second cycle of cryogelification. On the other hand, Figure 1c shows the second important thermal transition for the PVA. According to Sudhamani et al. [21] it corresponds to the water evaporation contained in the polymer dispersions. For heat flow, important differences between the samples were observed. Evaporation temperatures (*T*_e_) are different because they decrease depending on the increase in the number of cycles. The second cycle changes were minimal, indicating that more cryogelification cycles make difficult the evaporation of the water contained in the polymeric matrices. For the *C*p (Figure 1d), the third cycle was also the most relevant according to changes in the structure, as the number of cryogelification cycles increasing the three-dimensional conformation of the network was significantly modified [22].

**Figure 2:**
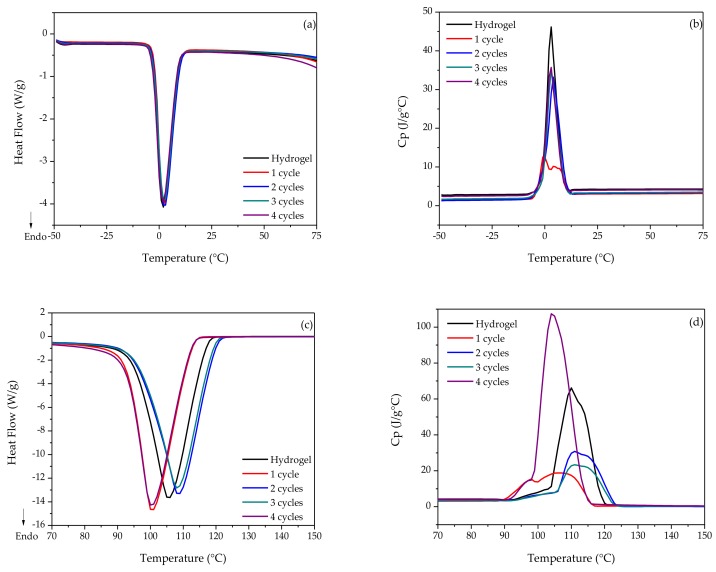
Thermal analysis of the hydro- and cryogels of WS with different freezing-thawing cycles: (**a**) heat flow during melting; (**b**) structural changes during fusion; (**c**) heat flow during evaporation; (**d**) structural changes during evaporation.

Figure 2a shows the total heat flow with the major changes occurring during the WS fusion. There were no significant changes since the *T*_m_ and the enthalpy were within the same range (Table S1). *C*p (Figure 2b) was modified by the effects of the structural arrangement of the amylopectin chain in order to generate a more ordered and stable three-dimensional network, except for the first cryogelification cycle. Figure 2c shows differences in the *T*_e_ in the evaporation zone of the WS polymer dispersions, which indicates that there are interactions of water with the polymers, reflected in the specific heat (Figure 2d), whereas the number of freeze–thaw cycles increases the changes in the structure. Lozinsky [15] reported that polymers spontaneously form gel networks at a certain rate and temperature below zero, and the kinetics of this process is strongly related to the condition of sub-cooling, as well as to the number of cryogelification cycles. Therefore, the formation of the gel network is controlled directly by kinetics, which can be described by its cryogelification cycles. In the case of WS, it presents a thermal phenomenon similar to PVA.

### 3.2. Structural Ordering Degree

The concept of order is related to the crystalline volume fraction in the dispersion, which indicates the structural order in the samples; consequently, there is a close relationship between physicochemical stability, changes in morphology and optical properties, and all of these are temperature dependent [23]. Table 1 shows that for the freeze–thaw cycles of polymers, values closer to 100% tend to possess a largely ordered structure. For the PVA case, the molecular arrangement of its structure occurred at four cycles, before a destabilization of the polymer chains occurred to form a three-dimensional network [24]. This phenomenon is closely related to the enthalpy values obtained by MDSC, since the four-cycle PVA has higher energy consumption, necessary to rearrange and stabilize the structure.

For the WS, the highest degree of order was achieved through two cryogelification cycles, corresponding to the greater enthalpy obtained by MDSC. This number of cycles was enough to rearrange the molecular structure to stabilize the system [23,25]. At the same time, Hulleman et al. [26], Jenkins and Donald [27], Lourdin et al. [28], and Van Soest et al. [29] define a close relationship between the rheological parameters and the structural ordering degree of the WS and its three-dimensional network formation. These properties affect its stability and functionality as a cryoprotectant material, resulting in enhanced rigidity.

### 3.3. SEM Analysis 

**Figure 3:**
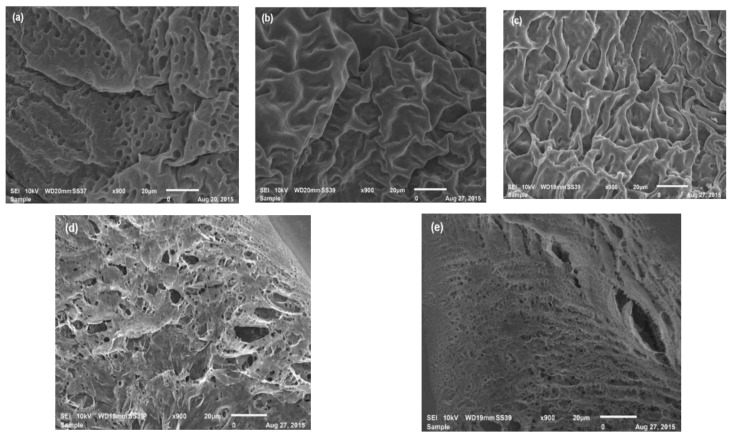
SEM micrographs (900×) of the PVA polymers: (**a**) hydrogel; (**b**) one-cycle cryogel; (**c**) two cycles; (**d**) three cycles; (**e**) four cycles.

Figure 3 shows that the polymer was affected by the number of cryogelification cycles. The pore size was smaller in the hydrogel and it increased from the first cryogelification cycle. At the third cycle, it presented a new alteration in the arrangement of the polymer network and formed a more homogeneous system that allowed it to capture small quantities of water between pores [30,31].

**Figure 4:**
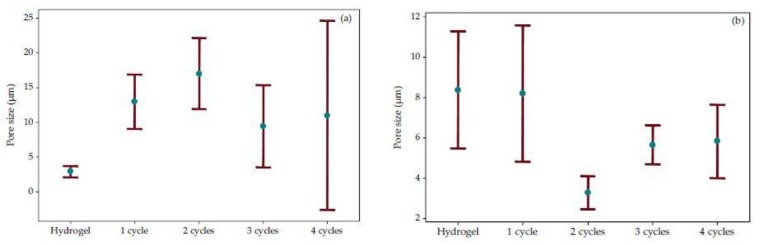
Confidence intervals at 95% of the polymer pore size: (**a**) PVA; (**b**) WS.

Figure 4a shows the distribution of the PVA pore sizes, showing that as the cryogelification cycles increase, they also enhance the heterogeneity of the pore sizes, which indicates that there are important structural arrangements corresponding with the MDSC analysis in this polymer. According to the structural ordering degree, cryogelification at four cycles did not present the smallest pore size, showing a greater dispersion of the data, which does not allow a control of the network spaces.

**Figure 5:**
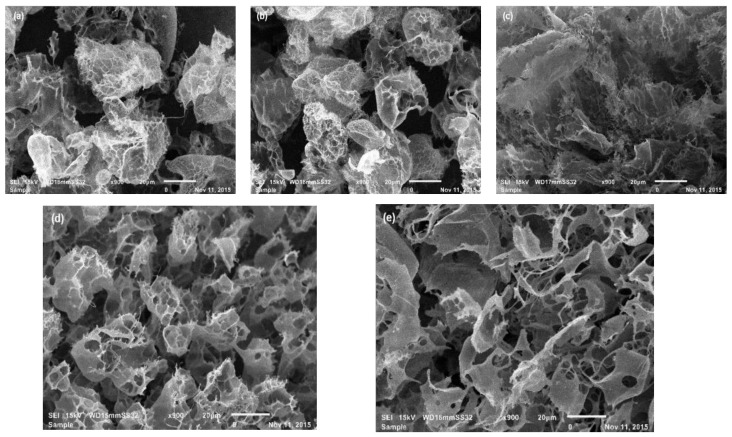
SEM micrographs (900×) of the WS polymers: (**a**) hydrogel; (**b**) one-cycle cryogel; (**c**) two cycles; (**d**) three cycles; (**e**) four cycles.

The WS micrographs (Figure 5) show aggregates of amylopectin different to the PVA. It does not form a traditional gel network, but small porous aggregates were reported by Huijbrechts et al. [32], Shi et al. [33], and Yoon et al. [34] where water is trapped. The amylopectin crystallization rate was approximately 0.5 times lower than for amylose, due to the inter- and intramolecular amylopectin chains formed during freezing [29].

Van Soest et al. [29] also reported that the ordering degree of the WS hydrogels will affect their mechanical properties, as well as the module, the resistance and the viscosity with a more ordered structure. The WS pore sizes are shown in Figure 4b, where the two-cycle cryogel presented a smaller value (2.44 ≤ μ ≤ 4.08), and at the same time, is more homogeneous with a lower data dispersion. This shows that this material has a viable structure for application in food processes.

### 3.4. Rheological Analysis

**Figure 6:**
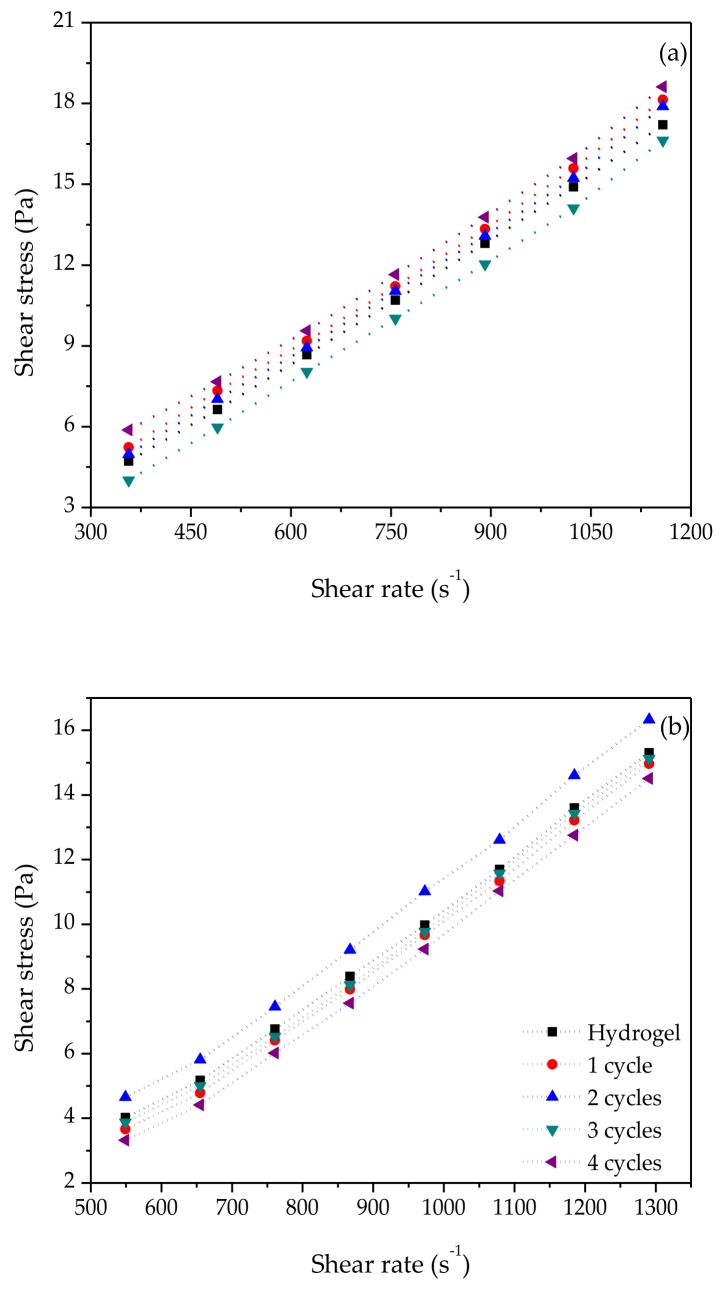
Rheological behavior of the polymers: (**a**) PVA; (**b**) WS.

The rheological characterization of PVA and WS at different cryogelification cycles indicates that the behavior of the dispersions was affected by the number of freeze–thaw cycles, fitting a Newtonian not-time-independent fluid behavior, following the Ostwald de Waele law, since the flow properties depend only on shear rate and are not constant in relation to the shear stress (Figure 6). In general, for the PVA case, minimal variations of the stress–shear curves depending on the number of freeze–thaw cycles were observed. However, for WS, the two-cycle cryogel showed a notable increase in the shear stress due to an increment in the shear rate (Figure 6b).

In both cases, the behavior was a shear thickening material, where the viscosity increases with the rise of the shear rate, due to a high concentration of particles that generate more collision and consequently more contact between them, as reported by Yang et al. [35] and Wang et al. [36]. This can be also observed for the rheological parameters (*n* and *k*), showing that for both polymers the *n* value was very close to 1 (Table 2), classifying these fluids as shear thickening.

### 3.5. FTIR Spectroscopy Analysis

**Figure 7:**
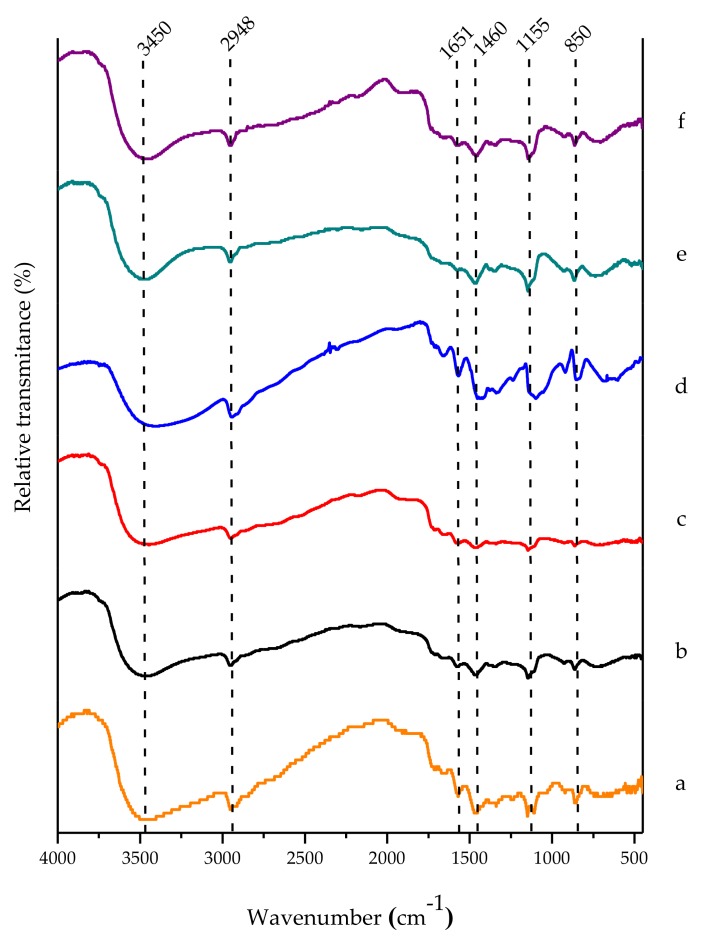
FTIR spectra of PVA hydro- and cryogels: (**a**) powder; (**b**) hydrogel; (**c**) one cycle; (**d**) two cycles; (**e**) three cycles; (**f**) four cycles.

Figure 7 shows the PVA FTIR-DR spectra; all the cases present bands at 3455–3450 cm^−1^ belonging to the stretching vibrations of OH^-^ groups, which correspond to the alcohol groups and water mainly in the structural form of dimers. The intensity of the band was not modified in any case, indicating that the association between water molecules was not affected by the cycles of cryogelification.

At 2948 cm^−1^, one can see stretches on the main aliphatic chain of the alkanes and alkyl groups (C–H) with moderately strong absorption intensities, which show no change in the function of the number of cryogelification cycles. At 1651 cm^−1^ an asymmetric stretching of the carbonyl groups (C=O) of the PVA acetate was observed. This band is also associated with C=C stretching of the terminal vinyl groups, decreasing its intensity in the hydrogel in the first, third and fourth freeze–thaw cycles, since they reduce the interactions between the vinyl groups and increase with water [33,37].

At 1460 cm^-1^ there was a combination of frequencies between the CH_3_–OH groups (interactions), showing asymmetric vibrations in the terminal methyl groups. These interactions were minor for one- and two-cycle cryogels in comparison to the other samples, indicating a close relationship with the increase in the pore size observed by SEM microscopy. At 1155 cm^−1^ stretches of the links between the C–O of tertiary alcohols were observed, as well as an asymmetric stretching of the C–O–C bonds of the aliphatic ethers. Finally, at 850 cm^−1^ there were bands of the CH_2_ terminal groups of the main chains, which are mostly emphasized with the two-cycle cryogel, closely related to the increase in the pore sizes observed by SEM studies [33,37,38].

**Figure 8:**
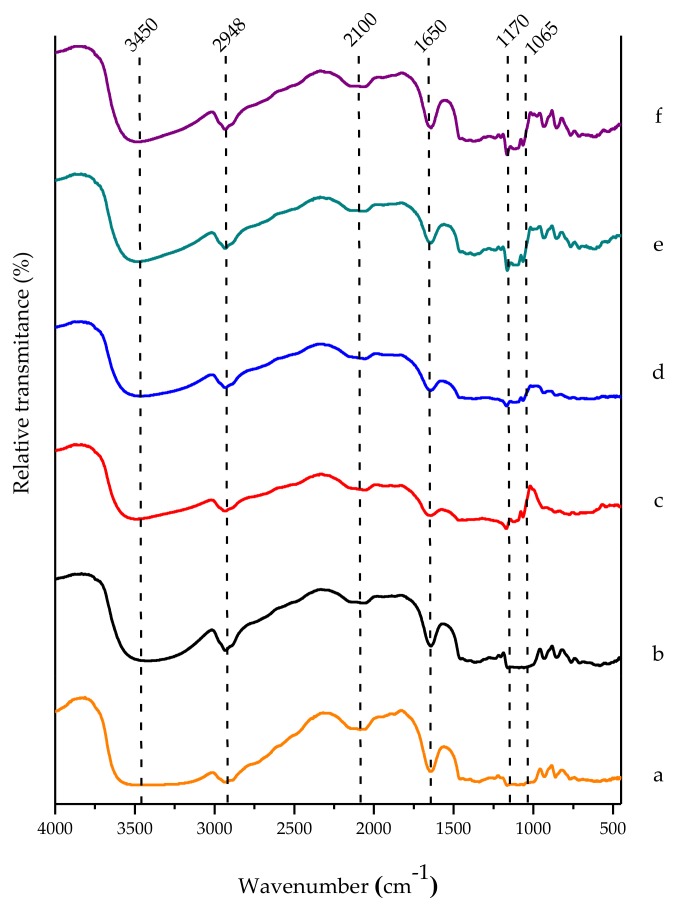
FTIR spectra of WS hydro- and cryogels: (**a**) powder; (**b**) hydrogel; (**c**) one cycle; (**d**) two cycles; (**e**) three cycles; (**f**) four cycles.

Besides, Figure 8 shows a band at 2100 cm^−1^ indicating stretching vibrations between C=C=O groups (ketenes) characteristic of natural polysaccharides. At 1650 cm^−1^, bending vibrations between O–H groups of the absorbed water in the amorphous regions of the starch were observed. This means that the band intensity decreases according to the increase of the cryogel’s order degree. For the two cryogelification cycles, this band was less intense. At 1170 cm^−1^, stretching vibrations of anhydroglucose rings were registered, which were modified from the first cryogelification cycle. At 1065 cm^−1^, the greater order degree in the cryogel bands intensity increased. Therefore, these bands appear from the first cycle of freezing, indicating that there are important structural arrangements affected by the freeze–thaw process that make the polymer system more stable according to the observed changes in the Cp in the MDSC studies [39,40,41].

## 4. Conclusions

It was demonstrated that significant changes were produced by the freeze–thaw cycles of the WS hydrogel. Two cryogelification cycles were sufficient to obtain a cryogel with physicochemical, thermodynamic and structural properties similar to those of PVA.

## Figures and Tables

**Table 1:** Structural order percentage of the hydro- and cryogel dispersions.

Sample	PVA	WS
Hydrogel	84.47 ± 0.52	84.12 ± 0.33
1 cycle	85.90 ± 0.97	90.80 ± 0.18
2 cycle	81.70 ± 0.92	94.72 ± 0.14
3 cycles	85.53 ± 1.17	89.05 ± 0.87
4 cycles	93.30 ± 1.11	89.41 ± 0.56

Mean ± standard deviation.

**Table 2:** Rheological parameters according to the Ostwald de Waele’s law.

Sample	PVA		WS	
	*n*	*k*	*n*	*k*
Hydrogel	0.9767 ± 0.0008	0.0183 ± 0.0007	1.4999 ± 0.0015	4 × 10^−4^ ± 0.0002
1 cycle	1.0425 ± 0.0011	0.0113 ± 0.0014	1.5855 ± 0.0009	2 × 10^−4^ ± 0.0001
2 cycles	1.0723 ± 0.0002	0.0091 ± 0.0003	1.6198 ± 0.0006	1 × 10^−4^ ± 0.0001
3 cycles	1.0973 ± 0.0010	0.0074 ± 0.0004	1.6696 ± 0.0012	1 × 10^−4^ ± 0.0001
4 cycles	1.1940 ± 0.0009	0.0036 ± 0.0002	1.7510 ± 0.0018	1 × 10^−5^ ± 0.00001

Mean ± standard deviation

## Supplementary Materials

The following are available online at http://www.mdpi.com/2073-4360/10/4/359/s1, Table S1: Enthalpies of melting and evaporation of the hydro- and cryogels.

## References

[1] Ciolacu D., Rudaz C., Vasilescu M., Budtova T. (2016). Phisically and Chemically cross-linked cellulose cryogels: Structure, properties and application for controlled release. Carbohydr. Polym..

[2] Gun’ko V.M., Savina I.N., Mikhalovsky S.V. (2013). Cryogels: Morphological, structural and adsorption characterization. Adv. Colloid Interface Sci..

[3] Regand A., Goff H.D. (2003). Structure and ice recrystallization in frozen stabilized ice cream model systems. Food Hydrocoll..

[4] Morris G.J., Acton E. (2013). Controlled ice nucleation in cryopreservation—A review. Cryobiology.

[5] Kamoun E.A., Chen X., Eldin M.S.M., Kenawy E.R.S. (2015). Crosslinked poly (vinyl alcohol) hydrogels for wound dressing applications: A review of remarkably blended polymers. Arabian J. Chem..

[6] Mansur H.S., Oréfice R.L., Mansur A.A.P. (2004). Characterization of poly (vinyl alcohol)/poly(ethylene glycol) hydrogels and PVA-derived hybrids by small-angle X-ray scattering and FTIR spectroscopy. Polymer.

[7] Patachia S., Dobritoiu R., Coviello T. (2011). Determination of the sorption efficiency of poly (vinyl alcohol)/scleroglucan cryogels, against Cu^+2^ ions. Env. Eng. Manag. J..

[8] Smith T.J., Kennedy J.E., Higginbotham C.L. (2009). The rheological and thermal characteristics of freeze-thawed hydrogels containing hydrogen peroxide for potential wound healing applications. J. Mech. Behav. Biomed. Mater..

[9] Lionetto F., Maffezzoli A., Ottenhof M.-A., Farhat I.A., Mitchell J.R. (2006). Ultrasonic investigation of wheat starch retrogradation. J. Food Eng..

[10] Zobel H. (1988). Starch crystal transformations and their industrial importance. Starch-Stärke.

[11] Van Hung P., Maeda T., Morita N. (2006). Waxy and high-amylose wheat starches and flours—characteristics, functionality and application. Trends Food Sci. Technol..

[12] Seetapan N., Limparyoon N., Fuongfuchat A., Gamonpilas C., Methacanon P. (2016). Effect of freezing rate and starch granular morphology on ice formation and non-freezable water content of flour and starch gels. Int. J. Food Prop..

[13] Yu X., Yu H., Zhang J., Shao S., Xiong F., Wang Z. (2015). Endosperm structure and physicochemical properties of starches from normal, waxy, and super-sweet maize. Int. J. Food Prop..

[14] Giannouli P., Morris E.R. (2003). Cryogelation of xanthan. Food Hydrocoll..

[15] Lozinsky V.I. (2002). Cryogels on the basis of natural and synthetic polymers: preparation, properties and application. Russ. Chem. Rev..

[16] Meléndez P.R., Arjona R.J.L., Velázquez C.R.R., Méndez A.A., Vázquez D.A. (2011). On the thermal properties of frozen, refrozen and freeze drying porcine *Longissimus dorsi*. J. Anim. Vet. Adv..

[17] Arjona R.J.L., Hernández G.R.P., Navarro L.I., Coria H.J., Rosas M.M.E., Meléndez P.R. (2016). Heat capacity prediction during pork meat thawing: Application of artificial neural network. J. Food Process Eng..

[18] Patel A.K., Bajpai R., Keller J.M. (2014). On the crystallinity of PVA/palm leaf biocomposite using DSC and XDR techniques. Microsyst. Technol..

[19] El-Sayed S., Mahmoud K.H., Fatah A.A., Hassen A. (2011). DSC, TGA and dielectric properties of carboxymethyl cellulose/polyvinyl alcohol blends. Physica B.

[20] Qi X., Hu X., Wei W., Yu H., Li J., Zhang J., Dong W. (2015). Investigation of salecan/poly(vinyl alcohol) hydrogels prepared by freeze/thaw method. Carbohydr. Polym..

[21] Sudhamani S.R., Prasad M.S., Udaya-Sankar K. (2003). DSC and FTIR studies on gellan and polyvinyl alcohol (PVA) blend films. Food Hydrocoll..

[22] Gómez I., Otazo E.M., Hernández H., Rubio E., Varela J., Ramírez M., Barajas I., Gordillo A.J. (2015). Thermal degradation study of PVA derivative with pendant phenylthionecarbamate groups by DSC/TGA and GC/MS. Polym. Degrad. Stab..

[23] Kong Y., Hay J.N. (2002). The measurement of the crystallinity of polymers by DSC. Polymer.

[24] Elliot S.R. (1993). Physics of Amorphous Materials.

[25] Ball S., Guan H.P., James M., Myers A., Keeling P., Mouille G. (1996). From glycogen to amylopectin: A model for the biogenesis of the plant starch granule. Cell.

[26] Hulleman S.H.D., Janssen F.H.P., Feil H. (1998). The role of water during plasticization of native starches. Polymer.

[27] Jenkins P.J., Donald A.M. (1995). The influence of amylose on starch granule structure. Int. J. Biol. Macromol..

[28] Lourdin D., Dellavalle G., Colonna P. (1995). Influence of amylose content on starch films and foams. Carbohydr. Polym..

[29] Van Soest J.J.G., Hulleman S.H.D., De Wit D., Vliegenthart J.F.G. (1996). Crystallinity in starch bioplastics. Ind. Crop. Prod..

[30] Chaturvedi A., Bajpai A.K., Bajpai J., Singh S.K. (2016). Evaluation of poly (vinyl alcohol) based cryogel-zinc oxide nanocomposites for possible applications as wound dressing materials. Mater. Sci. Eng..

[31] Aouada F.A., De-Moura M.R., Fernandes P.R.G., Rubira A.F., Muniz E.C. (2005). Optical and morphological characterization of polyacrylamide hydrogel and liquid crystal systems. Eur. Polym. J..

[32] Huijbrechts A.M.L., Desse M., Budtova T., Franssen M.C.R., Visser G.M., Boeriu C.G., Sudhölter E.J.R. (2008). Physicochemical properties of etherified maize starches. Carbohydr. Polym..

[33] Shi M., Chen Y., Yu S., Gao Q. (2013). Preparation and properties of RS III from waxy maize starch with pullulanase. Food Hydrocoll..

[34] Yoon H.S., Lee J.H., Lim S.T. (2009). Utilization for retrograded waxy maize starch gels as tablet matrix for controlled release of theophylline. Carbohydr. Polym..

[35] Yang X., Liu Q., Chen X., Yu F., Zhu Z. (2008). Investigation of PVA/WS-chitosan hydrogels prepared by combined γ-irradiation and freeze-thawing. Carbohydr. Polym..

[36] Wang Y., Lue A., Zhang L. (2009). Rheological behavior of waterbone polyurethane/starch aqueous dispersions during cure. Polymer.

[37] Bhunia T., Giri A., Nasim T., Chattopadhyay D., Bandyopadhyay A. (2013). Uniquely different PVA-xanthan gum irradiated membranes as transdermal diltiazem delivery device. Carbohydr. Polym..

[38] Fan L., Yang H., Yang J., Peng M., Hu J. (2016). Preparation and characterization of chitosan/gelatin/PVA hydrogel for wound dressings. Carbohydr. Polym..

[39] Lu H.W., Zhang L.M., Wang C., Chen R.F. (2011). Preparation and properties of new micellar drug carriers based on hydrophobically modified amylopectin. Carbohydr. Polym..

[40] Shalviri A., Liu Q., Abdekhodaie M.J., Wu X.Y. (2010). Novel modified starch-xanthan gum hydrogels for controlled drug delivery: Synthesis and characterization. Carbohydr. Polym..

[41] Zou W., Yu L., Liu X., Chen L., Zhang X., Qiao D., Zhang R. (2012). Effects of amylose/amylopectin ratio on starch-based superabsorbent polymers. Carbohydr. Polym..

