# Supplementary material for: Thermal, Structural, and Rheological Characterization of Waxy Starch as a Cryogel for Its Application in Food Processing
Source: Polymers (Basel). 2018 Mar 23;10(4):359. doi: 10.3390/polym10040359 (PMC6414860; doi:10.3390/polym10040359)
Supplement: Supplementary file 1 [file polymers-10-00359-s001.pdf]

Table S1. Enthalpies of melting and evaporation of the hydro- and cryogels.

**Sample**	**PVA**		**WS**	
	**ΔH_m (J/g)**	**ΔH_e (J/g)**	**ΔH_m (J/g)**	**ΔH_e (J/g)**
Hydrogel	311.60	1980.00	336.90	2154.00
1 cycle	323.80	2103.00	338.90	2200.00
2 cycles	302.30	1838.00	351.10	2279.00
3 cycles	311.50	1888.00	330.70	2100.00
4 cycles	345.00	2156.00	336.00	2042.00
